# Methods Applied to Assess Real‐World Effectiveness of Drugs: A Scoping Review

**DOI:** 10.1002/prp2.70244

**Published:** 2026-05-05

**Authors:** Benedikte Irene von Osmanski, Janne Petersen, Charlotte Thor Petersen, Andreas Høiberg Bentsen, Espen Jimenez‐Solem, Mikkel Zöllner Ankarfeldt

**Affiliations:** ^1^ Copenhagen Phase IV Unit, Department of Clinical Pharmacology and Center for Clinical Research and Prevention Copenhagen University Hospital Bispebjerg and Frederiksberg Copenhagen Denmark; ^2^ Signum Life Science Copenhagen Denmark; ^3^ Section of Biostatistics, Department of Public Health University of Copenhagen Copenhagen Denmark; ^4^ Amgros Copenhagen Denmark; ^5^ Department of Clinical Pharmacology Copenhagen University Hospital, Bispebjerg and Frederiksberg Copenhagen Denmark; ^6^ Institute of Clinical Medicine, University of Copenhagen Copenhagen Denmark

**Keywords:** drug effectiveness, methodological research, pharmacoepidemiology, real‐world data, real‐world evidence

## Abstract

Drug effectiveness evaluations are needed to bridge the gap between premarketing trials and clinical practice, but the specific methods used in such evaluations remain unclear. Therefore, this review aimed to identify pharmacoepidemiological effectiveness studies based on real‐world data (RWD) and categorize them based on the methods applied and the drugs and outcomes investigated. We searched PubMed and Embase for relevant records published in the period July–December 2019. Eligible studies: (i) were RWD‐based; (ii) evaluated one or multiple drug exposures, and (iii) included at least one effectiveness outcome (as primary outcome). Among 4820 identified records, 1129 passed title‐abstract screening, 200 were randomly selected for full‐text assessment, and 87 were ultimately included. Of these, 55 included > 1000 patients, 22 included > 10 000 patients, and 22 explicitly restricted to new users. Chemotherapy was the most common exposure (*n* = 32), all‐cause mortality the most prevalent outcome (*n* = 52), and non‐use of the exposure drug (*n* = 42) the most frequent comparator category. Survival models (*n* = 55) were the dominating statistical models, and 13 studies used only descriptive statistics. In conclusion, well‐known pharmacoepidemiological methods were applied in the included studies, and many had large study populations. Common limitations were use of simple descriptive statistics and absence of active comparators.

## 1 Introduction

Solid evidence on drug effectiveness [1, 2] is needed to ensure that the efficacy [1, 2] demonstrated under controlled conditions in premarketing trials translates into meaningful improvements in patient outcomes in clinical practice. Such real‐world evidence (RWE) of drug effectiveness is an important aspect of pharmacoepidemiology, but there is a limited overview of how established methodological approaches are applied in practice to generate evidence on drug effectiveness using real‐world data (RWD). Therefore, we undertook the present scoping review to identify methods used in drug effectiveness studies based on RWD to support evaluations of prescribed drugs and, thereby, rational treatment of patients.

The superiority of randomized controlled trials (RCTs) to estimate drug efficacy is widely accepted, and they constitute a cornerstone in drug assessments. However, it is also established that estimates derived from an RCT are lacking in external validity [2, 3, 4, 5]. This is due to multiple RCT characteristics, which deviate from clinical practice such as the restricted study population [4, 5, 6, 7, 8, 9, 10, 11], selection of physicians [2, 3], blinding and behavior of patients and staff, and the requirement of informed consent from participants [12]. Thus, to confirm the intended effects of a drug in clinical practice, RWE of drug effectiveness is needed. The conditions for filling in this need are improving as the amount of RWD is growing [13, 14]. Moreover, the interest in RWD based drug evaluations is increasing across regulatory agencies [15, 16], the pharmaceutical industry, and the health care systems. Three recent reviews found that RWE was widely used in marketing authorization applications to the European Medicines Agency (EMA) in 2018–2019 [17] and the U.S. Food and Drug Administration (FDA) in January 2019–June 2021 and, among other purposes, contributed with documentation on drug effectiveness [17, 18, 19]. Interestingly, the RWE provided in several applications to the FDA was found to be inadequate for assessment [18]. In line with this, common methodological weaknesses—such as insufficient analysis plans and risk of selection bias and confounding—of RWE on drug efficacy/effectiveness were identified in applications to EMA [19]. These findings emphasize the presence of methodological uncertainty in RWD‐based drug studies. This uncertainty may be particularly relevant to effectiveness evaluations, which are less well‐established compared to RWD‐based safety assessments [20].

In response to the methodological uncertainty and the increasing interest in drug effectiveness evaluations, the pharmacoepidemiological field would benefit from an overview of the methodologies used in these types of studies. The need for such an overview arises from the recognition that discerning between evaluations of safety and effectiveness is meaningful due to their differing objectives and implications. Safety studies primarily focus on identifying and minimizing potential adverse effects associated with drug use. The pharmacoepidemiological field emerged primarily in response to the need for such safety evaluations, recognizing the inherent limitations of premarketing RCTs to detect all adverse reactions, especially rare and delayed effects [21, 22]. On the contrary, effectiveness studies aim to assess how well a drug achieves its intended therapeutic outcomes in real‐world clinical settings [1]. This is a different type of research question, which may cause different considerations in relation to outcome metrics, comparators, and confounding, resulting in different methodological approaches.

On this background, we conducted the present study to provide an overview of methodological practices employed in RWD‐based drug effectiveness studies supporting the further establishment and future development of the field. Through a systematic literature search, we sought to collect a representative sample of drug effectiveness studies and categorize them according to applied methods and the drugs and outcomes investigated.

## 2 Methods

We applied a scoping review design [23, 24, 25, 26, 27] and reported the study in accordance with the Preferred Reporting Items for Systematic Reviews and Meta‐Analyses extension for Scoping Reviews (PRISMA‐ScR) [24]. Prior to the initiation of the screening process, a protocol was prepared and shared via the Open Science Framework (OSF) on March 20, 2022 [28]. The review has been conducted by our cross‐disciplinary team possessing competencies within various fields, including medicine, pharmacology, statistics, and pharmacoepidemiology.

### 2.1 Eligibility Criteria

In this review, we included RWD‐based studies published in the English language. Gray literature and short publication formats were excluded. Furthermore, the following eligibility criteria, organized according to population, exposure, comparator, outcome, and study design (PECOS) [29], were applied: (i) *Population*: Studies investigating humans were considered for inclusion. Eligibility was not restricted to studies investigating certain age or disease groups; (ii) *Exposure*: Eligible studies investigated at least one exposure that meets the EMA's definition of a medicinal product [30], excluding vaccines. See Table S1 for further details of the exposure criteria. (iii) *Comparator*: No criteria regarding the type of comparator were applied. Thus, both studies with active comparators (i.e., a different drug, dose, or treatment regimen) and studies with non‐initiator comparator groups were eligible; (iv) *Outcome*: For a record to meet the definition of an effectiveness study [31], at least one of the primary outcomes had to measure the desired effect of the drug. Therefore, we evaluated each outcome relative to the drug's indication and intended effects. Repurposing studies investigating potential novel therapeutic effects of established drugs were not included. Furthermore, an effectiveness outcome could measure either the direct effect or a derived desired effect, e.g., decreased blood pressure on antihypertensive treatment or lower risk of cardiovascular events following antihypertensive treatment. If an outcome could be perceived as both a measure of effectiveness and safety, e.g., mortality and hospitalization rates following chemotherapy, the study was considered eligible; (v) *Study design*: Eligibility was restricted to observational studies based on RWD meeting the following definition: “Real‐world data are the data relating to patient health status and/or the delivery of health care routinely collected from a variety of sources” [32]. Thus, we only included studies based on secondary use of data, i.e., studies using existing registries, databases, or electronic healthcare data. If other types of data were used in combination with RWD, the study was excluded.

### 2.2 Information Sources and Search Strategy

PubMed and Embase were used as information sources. The search string was developed in PubMed through an iterative process and subsequently adjusted to Embase. Both search strings are available in the Supporting Information (Tables S2 and S3). Filters for English language and publication in the six‐month period July 1, 2019–December 31, 2019, were applied. This period was chosen to achieve new publications while concurrently avoiding the COVID‐19 era. We considered the period sufficient to provide a sample of studies representative of the practices within RWD‐based effectiveness studies. The final searches were carried out in PubMed and Embase on March 16 and March 22, 2022, respectively. The literature review management software program Rayyan was used to handle records in the screening process [33].

### 2.3 Selection and Data Charting Process

Three reviewers (BO, MA, and CP) participated in the screening at the title‐abstract level. To ensure a higher inter‐reviewer agreement, conflicts and records assigned a ‘maybe’ were discussed following screening of the first 100 titles and abstracts (50 records screened by BO and MA, and 50 records screened by BO and CP). Subsequently, records were screened by two reviewers (BO and either MA or CP), and conflicts/maybes were discussed and resolved on an ongoing basis. When approximately 20% of the records had been screened by two reviewers, we changed practice to single‐reviewer screening (see Table S1 for a description of the validation process preceding the change of practice). The remaining records were screened by BO or MA. In cases of doubt, the record was given a maybe label and discussed with the other reviewer.

The title/abstract screening resulted in 1129 potentially eligible studies, of which we randomly selected 200 records to proceed for full text assessment using the sample function in R. Full text assessment and data extraction were done simultaneously in a process initiated by two pilot phases of the first 53 studies. Five reviewers (BO, CP, MA, JP, and ES) participated in the first phase, and three reviewers (BO, CP, and MA) participated in the second phase. A detailed description of both pilot phases is provided in Table S1. The remaining studies were handled by either reviewer BO or CP, followed by a targeted validation of the statistical data items by reviewer MA. Furthermore, a statistician (reviewer JP) looked into cases of doubt concerning statistical data items and supervised the following categorization of these items.

### 2.4 Data Items

We collected information on author, title, country of data origin, number of included patients, exposure(s), comparator(s), and outcomes from the included studies. Moreover, we extracted analysis characteristics, including statistical model(s), method(s) for confounder control, and variables controlled for in the study. In addition, we assessed whether the studies applied a new‐user design, as we consider this to be an important methodological parameter in pharmacoepidemiological studies. The items are specified in Table S4. When studies reported multiple exposures, comparators, outcomes, or statistical models, data were extracted at the study level rather than for each individual exposure‐outcome analysis. Each specific item (e.g., exposure or statistical model) was therefore recorded only once per study, regardless of how many times it was applied within that study. Consequently, the number of statistical models does not correspond to the number of exposure–outcome analyses. Methods to control for confounding were extracted in connection to statistical models to allow for linkage between these two data items and could therefore be extracted more than once per study.

### 2.5 Data Synthesis

Results are presented as counts and proportions. The proportions can be a percentage of the study count (data origin, number of patients, new user design, variables controlled for) or a percentage of the specific item count (exposure drug, comparator category, outcome category, statistical models, methods to control for confounding). Following charting and validation, the data handling and descriptive statistics was conducted in SAS Enterprise Guide 7.1. Both the sunburst plot and the combination chart were created in Python 3.7.7 using the matplotlib package.

## 3 Results

**FIGURE 1:**
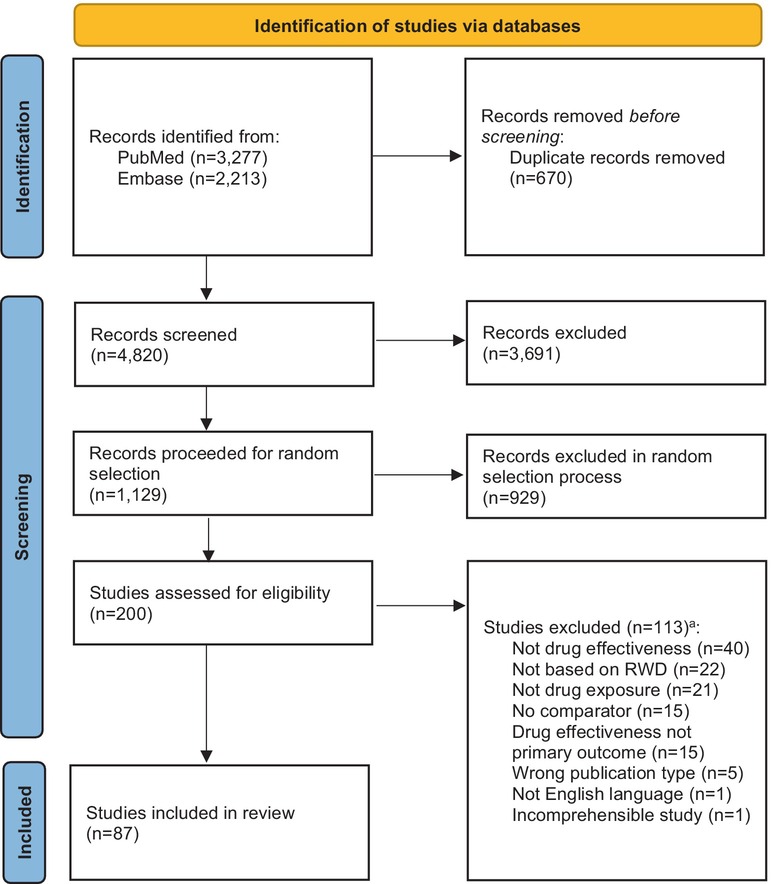
Flow diagram of the study selection process. ^a^Each study could be assigned more than one reason of exclusion. The figure is adapted from Page MJ, McKenzie JE, Bossuyt PM, Boutron I, Hoffmann TC, Mulrow CD, et al. The PRISMA 2020 statement: An updated guideline for reporting systematic reviews. BMJ 2021;372:N71. doi:10.1136/bmj.n7152 [34].

After removing the duplicates, 4820 unique records were retrieved from the literature searches. Of these, 1129 studies were found potentially eligible during screening of titles and abstracts. In the further selection process, 200 studies were randomly selected for further assessment, of which 87 were definitively included. The selection process is illustrated in a PRISMA diagram in Figure 1, and a complete reference list of the included studies is provided in Table S5.

Of the 87 included studies, 44 (50.6%) were based on data from the US, 21 (24.1%) from European countries, 13 (14.9%) from Asian countries, 5 (5.8%) from other countries, and 4 (4.6%) from multiple countries. Almost two‐thirds of the studies included more than 1000 patients (*n* = 55, 63.2%), and a quarter of the studies included more than 10 000 patients (*n* = 22, 25.3%). Data nationality, number of participants, and other key characteristics of the included studies are summarized in Table 1. The drug exposures in the included studies according to level two in the ATC classification system are listed in Table S6, showing that chemotherapy (ATC: L01) was the most frequent drug exposure investigated in 32 studies, while antithrombotic agents (ATC: B01) were the second most frequent drug exposure investigated in 13 studies. The remaining ATC groups were identified in six or fewer studies. Because chemotherapy (ATC: L01) was the dominating drug exposure, the study characteristics (as shown in Table 1) are listed separately for studies investigating the effectiveness of chemotherapy and studies investigating other drugs in Table S7.

**TABLE 1:** Key characteristics of the 87 included studies.

Study characteristics	Studies, *n* (%)
Area of data origin	
US	44 (50.6)
Europe	21 (24.1)
Asia	13 (14.9)
Other	5 (5.8)
Multinational	4 (4.6)
Number of patients	
1–100	6 (6.9)
101–1000	26 (29.9)
1001–10 000	33 (37.9)
10 001–100 000	19 (21.8)
> 100 000	3 (3.5)
Comparator category (*n* = 93)^a^	
Non‐use of exposure drug	42 (45.2)
Drug comparator	35 (37.6)
Different dose/admin. of the same drug	11 (11.8)
Non‐drug comparator	5 (5.3)
New user design	
No	65 (74.7)
Yes	22 (25.3)
Outcome category (*n* = 176)^a^	
Mortality/survival: All‐cause	52 (29.6)
Disease specific measures	29 (16.5)
Diagnosis	22 (12.5)
Surgery and procedures	17 (9.7)
Health care utilization	12 (6.8)
Mortality/survival: Cause specific	10 (5.7)
Hospital admission: All‐cause	9 (5.1)
Drug prescription/discontinuation/switch	8 (4.6)
Costs: Overall	6 (3.4)
Hospital admission: Cause specific	6 (3.4)
Costs: Disease/treatment specific	5 (2.8)
Statistical model category (*n* = 96)^a^	
Survival models	55 (57.3)
Regression models	25 (26.0)
Other models	16 (16.7)
Confounder control methods (*n* = 137)^a^	
Adjustment	52 (38.0)
Stratification	36 (26.3)
Propensity score matching	22 (16.1)
IPTW	13 (9.5)
Matching	3 (2.2)
Propensity score adjustment	3 (2.2)
Propensity score stratification	2 (1.5)
Stabilized inverse probability treatment weighting	2 (1.5)
Full optimal matching	1 (0.7)
High‐dimensional propensity score model with IPTW	1 (0.7)
Instrumental variable	1 (0.7)
Standardized mortality ratio weighting	1 (0.7)
Number of studies controlling for	
Demographic variables	67 (77.0)
Comorbidity	48 (55.2)
Disease severity	56 (64.4)
Social variables	19 (21.8)

*Note:* Key characteristics of the 87 included studies.

Abbreviation: IPTW, inverse probability of treatment weighting.

^a^ Because more than one category could be extracted from each study the numbers sum up to more than the 87 included studies. Each category could only be counted once per study.

In the data charting process, comparators and outcomes were categorized as shown in Table 1. Among the 87 studies, we identified four different comparator categories summing up to a count of 93, and 11 outcome categories summing up to a count of 176. The most frequent comparator category was non‐use of the exposure drug (*n* = 42), i.e., patients treated with the exposure drug are compared with patients not treated with that drug and not, e.g., an active comparator. The most frequent outcome category was all‐cause mortality/survival, which was identified in 52 studies. The least common outcome category was disease/treatment specific costs, which were observed in five studies.

We also collected information on the application of a new user design and found that 22 studies restricted their analyses to new users. In the remaining 65 studies, a new user design was either not applied or the information provided by the authors was not sufficient to conclude whether prevalent users were excluded or not.

**FIGURE 2:**
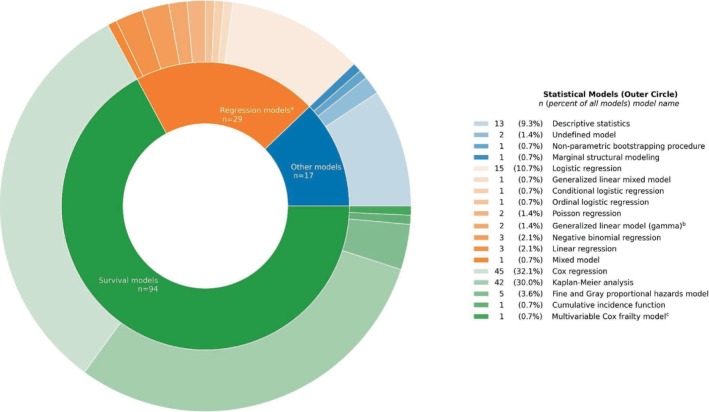
Sunburst plot of statistical models applied in the included studies. The models are categorized as survival, regression, or other models. ^a^Regression models include both general and generalized linear models. ^b^Generalized linear model with gamma distribution. ^c^Multivariable Cox frailty model with random intercepts.

The statistical models applied in the included studies to assess associations between drug exposures and effectiveness outcomes are listed at the category level in Table 1. Summing up to a count of 96, we observed survival models in 55 studies, regression models in 25 studies, and other models in 16 studies. Across the three model categories, the specific statistical models summed up to a count of 140, which are illustrated hierarchically in Figure 2. Cox regression (*n* = 45) and Kaplan–Meier analysis (*n* = 42) were the most common survival models, while logistic regression (*n* = 15) was the most frequently used regression model. It should be noted that descriptive statistics were included in the overview of statistical models but only for studies applying no other models. As illustrated in Figure 2, 13 studies applied descriptive statistics only.

**FIGURE 3:**
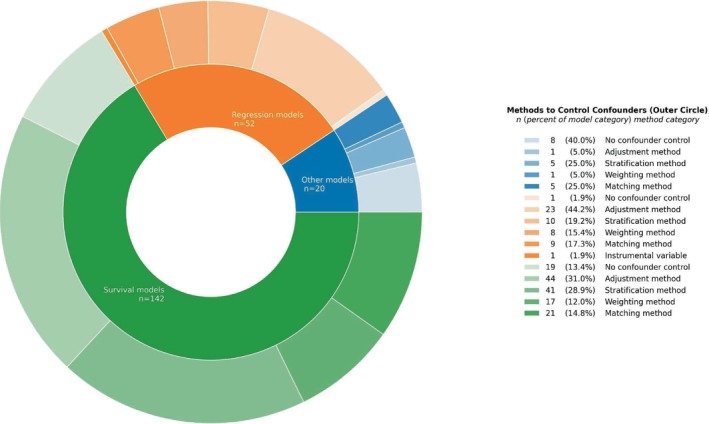
Sunburst plot illustrating the frequency of combinations between counter‐confounding method categories and statistical model categories. The count of combinations (*n* = 214) exceeds the count of statistical models (*n* = 140), as each statistical model could be combined with more than one counter‐confounding method per study.

We extracted different methods to control for confounding as listed in Table 1. Among the 87 studies, the methods to control for confounding summed up to a count of 137, with adjustment by multivariable models (*n* = 52), stratification (*n* = 36), and propensity score matching (*n* = 22) being the most frequently applied methods. All the specific methods to counter confounding identified in the studies were categorized depending on whether the methods were based on adjustment, weighting, matching, stratification, or instrumental variables. These five categories are depicted in Figure 3, which illustrates the frequency of combinations between the counter‐confounding methods and the overall categories of statistical models. As seen in Figure 3, both regression and survival models were most frequently combined with adjustment, stratification, and matching‐based methods. In addition to charting the counter‐confounding methods applied, we also summarized which types of variables the studies controlled for in their analyses. As listed in Table 1, the majority of the 87 included studies controlled for demographic variables (*n* = 67), while fewer studies controlled for comorbidity (*n* = 48) and disease severity (*n* = 56), and a minority of studies controlled for social variables (*n* = 19).

## 4 Discussion

### 4.1 Key Findings

The present scoping review aimed at mapping applied methods, outcomes, and the drugs investigated among RWD‐based drug effectiveness studies. In the 87 included studies, we found that one third of the studies investigated chemotherapy, half of the comparators were non‐use of the exposure drug, a quarter of the studies included more than 10 000 participants, and few studies applied a new‐user design. Survival and regression models were the most frequently applied statistical models, and 13 studies applied descriptive statistics only. To counter confounding in the analyses, the regression and survival models were most frequently applied in combination with adjustment, stratification, and matching based methods. Jointly, these findings indicate that methods used in observational drug effectiveness studies are well‐established and familiar, but also that the important new‐user, active comparator design [35] was infrequently used, and many studies did not include confounder adjustment for comorbidity, disease severity, and social variables.

### 4.2 Data and Design

With survival and regression models being the most applied statistical models across the included studies and multivariable adjustment and stratification being the most frequently employed techniques to mitigate confounding, our findings indicate that researchers of observational drug effectiveness studies reach towards well‐established methods found in the pharmacoepidemiological toolbox. While the frequent use of survival models was expected, given the common use of time‐to‐event outcomes, particularly in studies evaluating chemotherapy, this finding nevertheless documents applied methodological practice in RWD‐based drug effectiveness research. Notably, survival models were frequently applied beyond chemotherapy studies, suggesting that their use is not solely driven by drug class but may also reflect the widespread availability of mortality and time‐to‐event outcomes in routinely collected healthcare data. This is in line with our findings related to applied outcomes, where all‐cause mortality/survival was the most frequently observed outcome category—a finding which is constituent with a previous study from our group mapping outcome measures in Danish pharmacoepidemiological studies, including both safety and effectiveness studies [36].

Other than reflecting the widespread availability of mortality measures in RWD sources, the frequent use of mortality outcomes also reflects that death can be a very relevant effectiveness outcome in chemotherapy studies, which constitute a considerable part of the included studies. If focusing on studies investigating non‐chemo drugs, we found that many studies included more specific outcome measures such as disease‐specific measures, diagnoses, and surgery and procedures. The availability of more specific outcome measures is of great importance both to the clinical value of pharmacoepidemiological studies but also to the comparability of the results with previous findings from other observational studies and RCTs. Surprisingly, we found that cost‐related outcomes were rarely utilized despite their clinical importance as premarketing RCTs often fail to predict them. The finding aligns with our previous review, where cost measures appeared in less than < 1% of the studies [36]. Although costs do not directly measure drug effectiveness, they offer valuable insights by reflecting health care needs and, indirectly, patient health status. Therefore, we included cost measures as effectiveness outcomes in the present study.

In relation to the identified outcomes, one could argue that differentiating between outcomes of safety and effectiveness is arbitrary or lacks methodological significance compared to other outcome parameters such as frequency or time to event. Moreover, the differentiation is challenging, especially due to overlapping outcome metrics that may apply to both safety and effectiveness, such as hospitalization rates. However, we wanted to generate knowledge of the methods used in effectiveness studies and therefore needed to discern between studies of safety and effectiveness because both the research questions and the clinical interpretation of the findings differ.

New and innovative statistical methods to advance pharmacoepidemiology into the area of studying intended drug effects were not identified. Furthermore, 13 out of the 87 studies fulfilling the inclusion criteria applied descriptive statistics only. Given the limited inferential capacity of descriptive results, the validity of conclusions drawn on drug effectiveness from such studies is limited. The same doubt may apply to studies where descriptive statistics were only supplemented by Kaplan–Meier analyses. However, to gain insights into the breadth of pharmacoepidemiological studies, we did not set up any eligibility criteria regarding the type of statistical analyses applied in the studies, except that they were required to include a comparator group.

Looking into the findings regarding the four categories of variables used for confounder adjustment (demographics, comorbidity, disease severity, and social variables), each of them was absent in a considerable number of the studies. The absence ranged from 20 studies (demographics) to 68 studies (social variables). Due to the lack of key variables in the analyses, confounding may affect the results in a considerable number of studies. As we find it unlikely that researchers would exclude important variables from their analyses, we believe that these findings reflect the extent of the data available. This is in line with a study of RWE on drug effectiveness included in submissions to the FDA, which found that the included RWD were often insufficient to serve the purpose of demonstrating drug effects [20].

We observed that some studies did not investigate specific drugs but used overall drug classes such as ‘chemotherapy’ instead. This indicates clear inferential limitations of these studies' results. One possible explanation for the absence of information on specific drugs is limitations inherent in the available data sources, as mentioned in some of the included studies [37, 38, 39]. While this finding highlights a common limitation of RWE, our findings regarding the patient numbers in the 87 studies illustrates a common strength—pharmacoepidemiological studies often include more patients than is feasible in RCTs.

Only a minority of the included studies clearly implemented a new‐user design. Investigating prevalent users can lead to bias, and the new‐user design is widely regarded as a benchmark for quality in pharmacoepidemiology, including comparative effectiveness research [35, 40].

### 4.3 Limitations

Our cross‐disciplinary review team has secured a high level of knowledge from both the medical, pharmacological, statistical, and pharmacoepidemiological field to guide the decision‐making during the study conduct. Still, some important limitations should be mentioned. Firstly, we chose to limit our literature search to a six‐month period to obtain a reasonable number of records for screening. Moreover, to match our previous work [36] and to avoid the possible effects on focus and study design by the covid‐19 pandemic era, we chose to search for studies from the second half of 2019. Even though we consider this an advantage of our study, we cannot exclude that it has reduced the extent to which our findings reflect current practice in pharmacoepidemiologic effectiveness studies. However, we do not believe that our findings would change considerably if a newer or longer search period was used. Secondly, some of our eligibility criteria were challenging to assess. For example, it was often necessary to carefully evaluate the study text to assess whether the data sources used met the definition of RWD. Likewise, some study assessments led to discussion regarding our definition of an effectiveness study, and we ended up including these studies [41, 42] even though their primary purpose was not clearly presented. Altogether, the challenges related to assessment of eligibility may have affected the reproducibility of our findings. However, involving up to five reviewers in reaching consensus in the study selection process, we believe the included studies to represent drug effectiveness studies. Thirdly, when extracting data items, we did not rate the extent to which the methods were applied. E.g., a study was categorized as controlling for demographic variables irrespective of the number of demographic variables included in the analysis. Fourthly, we deviated from our protocol in the screening and data charting processes, as these were only partially carried out independently by two reviewers. As described in our methods, these deviations were preceded by validation processes to secure the quality of our work. Lastly, only a subset of 200 records proceeded from title‐abstract screening to full text assessment in the selection process. As the selection was random, we believe our sample to be representative of the studies.

### 4.4 Perspectives

The experiences and findings from the present study point to several future research directions and priority areas for RWD–based drug effectiveness evaluations. In particular, further investigation into the use of fit‐for‐purpose data is warranted. This could be addressed in future reviews assessing the data sources applied in RWD‐based drug effectiveness studies within one or a limited number of specific therapeutic areas. Such reviews could combine quantitative summaries of data source use with qualitative assessments of whether the applied data are fit for purpose, including their ability to provide valid and sufficiently detailed measures of exposures, outcomes, and confounders. Similarly, qualitative assessments could evaluate the risk of selection bias and the suitability of the applied statistical methods in relation to the research questions and available data.

Although insufficient reporting is a well‐described concern within epidemiological research [43], and the medical literature in general [44], the extent of reporting issues struck us along the review process. We observed a lack of transparency in study reporting concerning data sources, definitions of treatments, outcomes and how statistical analyses were performed. This is problematic, as inadequate reporting of, e.g., data sources affects the readers access to assess whether a study is based on fit for purpose data. Naturally, insufficient reporting does not apply to all studies, and we also observed many well‐reported studies throughout this review. However, we believe these issues to be an important point of action in future research.

## 5 Conclusion

In this review, we investigated exposure drugs, outcomes, and applied methods in 87 pharmacoepidemiological effectiveness studies. We found that the included studies applied well‐established pharmacoepidemiological methods and statistical analyses. However, we also found that studies were limited by using simple descriptive statistics, no active comparator, and many studies were limited by data availability. Future pharmacoepidemiological effectiveness studies would benefit from an increased focus on explicit reporting of data sources and applied methods e.g., by using reporting guidelines. We believe that RWE studies on drug effectiveness have the potential to contribute with important clinical evidence, and we encourage relevant stakeholders to increase their focus on this area of evidence for the benefit of patients and health care systems. However, realizing this potential requires not only access to high‐quality RWD sources but also initiatives to support the necessary methodological evaluation and development. Addressing these issues will help generate evidence to guide clinical and regulatory decisions and support rational use of drugs.

## Author Contributions

**Benedikte Irene von Osmanski:** conceptualization, investigation, writing – original draft, methodology, validation, visualization, formal analysis, project administration, data curation. **Janne Petersen:** conceptualization, investigation, funding acquisition, methodology, validation, visualization, writing – review and editing, formal analysis, supervision, data curation. **Charlotte Thor Petersen:** investigation, validation, writing – review and editing. **Andreas Høiberg Bentsen:** investigation, writing – review and editing, supervision. **Espen Jimenez‐Solem:** conceptualization, investigation, funding acquisition, methodology, validation, writing – review and editing, supervision. **Mikkel Zöllner Ankarfeldt:** conceptualization, investigation, funding acquisition, methodology, validation, visualization, writing – review and editing, formal analysis, supervision, data curation.

## Funding

This work was supported by Innovationsfonden.

## Conflicts of Interest

The authors would like to disclose the following industrial relationship not directly related to the present work: **Mikkel Zöllner Ankarfeldt** has participated in research projects funded by Eli Lilly, Gilead, Novartis, Johnson & Johnson, and Vertex Pharmaceuticals. All funds were given to his institution. **Janne Petersen** has participated in research projects funded by Eli Lilly, Johnson & Johnson, Amgen, Novo Nordisk, Gilead, Novartis, and Vertex Pharmaceuticals. All funds were given to her institution. **Espen Jimenez‐Solem** has participated in research projects funded by Eli Lilly, Johnson & Johnson, UCB, Gilead, Novartis, and Vertex Pharmaceuticals. All funds were given to his institution. **Benedikte Irene von Osmanski**, **Charlotte Thor Petersen**, and **Andreas Høiberg Bentsen** have nothing to disclose.

## Supporting information


**Table S1:** Elaboration of the methods section.
**Table S2:** PubMed search string and results as of 16MAR2022.
**Table S3:** Embase search string and results as of 22MAR2022.
**Table S4:** Data items collected and used in the data synthesis.
**Table S5:** List of the 87 included studies.
**Table S6:** Frequency of studies with specific ATC groups.
**Table S7:** Key characteristics of the 87 included studies divided in studies investigating the effectiveness of chemotherapy and studies investigating the effectiveness of other drugs.

## Data Availability

The data that support the findings of this study are available from the corresponding author upon reasonable request.

## References

[1] B. L. Strom , S. E. Kimmel , and S. Hennessy , “Views From Academia, Industry, Regulatory Agencies, and the Legal System,” in Textbook of Pharmacoepidemiology, 3rd ed. (John Wiley & Sons, 2022), 76.

[2] B. Haynes , “Can It Work? Does It Work? Is It Worth It?,” BMJ 319 (1999): 652–653. 10480802 10.1136/bmj.319.7211.652 PMC1116525

[3] S. Saturni , F. Bellini , F. Braido , et al., “Randomized Controlled Trials and Real Life Studies. Approaches and Methodologies: A Clinical Point of View,” Pulmonary Pharmacology & Therapeutics 27 (2014): 129–138, 10.1016/j.pupt.2014.01.005. 24468677

[4] J. R. Bosdriesz , V. S. Stel , M. van Diepen , et al., “Evidence‐Based Medicine—When Observational Studies Are Better Than Randomized Controlled Trials,” Nephrology (Carlton) 25 (2020): 737–743, 10.1111/nep.13742. 32542836 PMC7540602

[5] H. G. C. Van Spall , A. Toren , A. Kiss , et al., “Eligibility Criteria of Randomized Controlled Trials Published in High‐Impact General Medical Journals: A Systematic Sampling Review,” JAMA 297 (2007): 1233–1240, 10.1001/jama.297.11.1233. 17374817

[6] D. C. Grootendorst , K. J. Jager , C. Zoccali , et al., “Observational Studies Are Complementary to Randomized Controlled Trials,” Nephron. Clinical Practice 114 (2010): c173–7. 19955822 10.1159/000262299

[7] K. R. Bailey , “Generalizing the Results of Randomized Clinical Trials,” Controlled Clinical Trials 15 (1994): 15–23, 10.1016/0197-2456(94)90024-8. 8149769

[8] R. Dowd , R. R. Recker , and R. P. Heaney , “Study Subjects and Ordinary Patients,” Osteoporosis International 11 (2000): 533–536, 10.1007/s001980070097. 10982170

[9] L. Wei , S. Ebrahim , C. Bartlett , P. D. Davey , F. M. Sullivan , and T. M. MacDonald , “Statin Use in the Secondary Prevention of Coronary Heart Disease in Primary Care: Cohort Study and Comparison of Inclusion and Outcome With Patients in Randomised Trials,” BMJ 330 (2005): 821, 10.1136/bmj.38398.408032.8F. 15790616 PMC556073

[10] P. Y. Lee , K. P. Alexander , B. G. Hammill , et al., “Representation of Elderly Persons and Women in Published Randomized Trials of Acute Coronary Syndromes,” Jama 286 (2001): 708–713, 10.1001/jama.286.6.708. 11495621

[11] J. J. V. McMurray and E. O'Meara , “Treatment of Heart Failure With Spironolactone — Trial and Tribulations,” New England Journal of Medicine 351 (2004): 526–528, 10.1056/NEJMp048144. 15295043

[12] J. F. Bergmann , O. Chassany , J. Gandiol , et al., “A Randomised Clinical Trial of the Effect of Informed Consent on the Analgesic Activity of Placebo and Naproxen in Cancer Pain,” Clinical Trials and Meta‐Analysis 29 (1994): 41–47. 10150184

[13] W. H. Crown , “Real‐World Evidence, Causal Inference, and Machine Learning,” Value in Health 22 (2019): 587–592, 10.1016/j.jval.2019.03.001. 31104739

[14] M. Schmidt , S. A. J. Schmidt , J. L. Sandegaard , V. Ehrenstein , L. Pedersen , and H. T. Sørensen , “The Danish National Patient Registry: A Review of Content, Data Quality, and Research Potential,” Clinical Epidemiology 7 (2015): 449–490, 10.2147/CLEP.S91125. 26604824 PMC4655913

[15] U.S. Food & Drug Administration , [Homepage on the Internet]“Framework for FDA'S Real‐World Evidence Program,” n.d.

[16] European Medicines Agency (EMA) [Homepage on the Internet] , “Data Analysis and Real World Interrogation Network (DARWIN EU),” n.d accessed January 20, 2025, https://www.ema.europa.eu/en/about‐us/how‐we‐work/big‐data/data‐analysis‐real‐world‐interrogation‐network‐darwin‐eu.

[17] R. Flynn , K. Plueschke , C. Quinten , et al., “Marketing Authorization Applications Made to the European Medicines Agency in 2018–2019: What Was the Contribution of Real‐World Evidence?,” Clinical Pharmacology and Therapeutics 111 (2022): 90–97, 10.1002/cpt.2461. 34689339 PMC9299056

[18] C. A. Purpura , E. M. Garry , N. Honig , et al., “The Role of Real‐World Evidence in FDA‐Approved New Drug and Biologics License Applications,” Clinical Pharmacology & Therapeutics 111 (2022): 135–144, 10.1002/cpt.2474. 34726771 PMC9299054

[19] E. Bakker , K. Plueschke , C. J. Jonker , X. Kurz , V. Starokozhko , and P. G. M. Mol , “Contribution of Real‐World Evidence in European Medicines Agency's Regulatory Decision Making,” Clinical Pharmacology & Therapeutics 113 (2023): 135–151, 10.1002/cpt.2766. 36254408 PMC10099093

[20] N. Mahendraratnam , K. Mercon , M. Gill , L. Benzing , and M. B. McClellan , “Understanding Use of Real‐World Data and Real‐World Evidence to Support Regulatory Decisions on Medical Product Effectiveness,” Clinical Pharmacology and Therapeutics 111 (2022): 150–154, 10.1002/cpt.2272. 33891318

[21] D. H. Lawson , “Pharmacoepidemiology: A New Discipline,” British Medical Journal (Clinical Research ed.) 289 (1984): 940–941. 6435731 10.1136/bmj.289.6450.940 PMC1443195

[22] P. Turner , “Long‐Term Assessment of Drug Safety and Efficacy,” Journal of the Royal Society of Medicine 77 (1984): 93–94. 6737400 10.1177/014107688407700201 PMC1439688

[23] Z. Munn , M. D. J. Peters , C. Stern , C. Tufanaru , A. McArthur , and E. Aromataris , “Systematic Review or Scoping Review? Guidance for Authors When Choosing Between a Systematic or Scoping Review Approach,” BMC Medical Research Methodology 18 (2018): 143, 10.1186/s12874-018-0611-x. 30453902 PMC6245623

[24] A. C. Tricco , E. Lillie , W. Zarin , et al., “PRISMA Extension for Scoping Reviews (PRISMA‐ScR): Checklist and Explanation,” Annals of Internal Medicine 169 (2018): 467–473, 10.7326/M18-0850. 30178033

[25] M. D. J. Peters , C. Marnie , A. C. Tricco , et al., “Updated Methodological Guidance for the Conduct of Scoping Reviews,” JBI Evidence Synthesis 18 (2020): 2119–2126, 10.11124/JBIES-20-00167. 33038124

[26] M. T. Pham , A. Rajic , J. D. Greig , et al., “A Scoping Review of Scoping Reviews: Advancing the Approach and Enhancing the Consistency,” Research Synthesis Methods 5 (2014): 371–385, 10.1002/jrsm.1123. 26052958 PMC4491356

[27] A. C. Tricco , J. Antony , W. Zarin , et al., “A Scoping Review of Rapid Review Methods,” BMC Medicine 13 (2015): 224, 10.1186/s12916-015-0465-6. 26377409 PMC4574114

[28] B. I. von Osmanski , M. Z. Ankarfeldt , J. Petersen , et al., “Available Pharmacoepidemiological Methods to Assess Real‐World Effectiveness of Drugs: A Scoping Review Protocol,” (2022), 10.17605/OSF.IO/XB2R5.

[29] R. L. Morgan , P. Whaley , K. A. Thayer , and H. J. Schünemann , “Identifying the PECO: A Framework for Formulating Good Questions to Explore the Association of Environmental and Other Exposures With Health Outcomes,” Environment International 121 (2018): 1027–1031, 10.1016/j.envint.2018.07.015. 30166065 PMC6908441

[30] European Medicines Agency (EMA) [Homepage on the Internet] , “Medicinal Product,” n.d. accessed January 20, 2025, https://www.ema.europa.eu/en/glossary‐terms/medicinal‐product.

[31] B. L. Strom , S. E. Kimmel , and S. Hennessy , “Appendix B: Glossary,” in Textbook of Pharmacoepidemiology, 3rd ed. (John Wiley & Sons, 2022), 496.

[32] U.S. Food & Drug Administration [Homepage on the Internet] , “Real‐World Evidence,” (2024) accessed January 20, 2025, https://www.fda.gov/science‐research/science‐and‐research‐special‐topics/real‐world‐evidence.

[33] M. Ouzzani , H. Hammady , Z. Fedorowicz , et al., “Rayyan—A Web and Mobile App for Systematic Reviews,” Systematic Reviews 5 (2016), 10.1186/s13643-016-0384-4. PMC5139140 27919275

[34] M. J. Page , J. E. McKenzie , P. M. Bossuyt , et al., “The PRISMA 2020 Statement: An Updated Guideline for Reporting Systematic Reviews,” BMJ (Clinical Research ed.) 372 (2021): n71, 10.1136/bmj.n71. PMC8005924 33782057

[35] J. L. Lund , D. B. Richardson , and T. Stürmer , “The Active Comparator, New User Study Design in Pharmacoepidemiology: Historical Foundations and Contemporary Application,” Current Epidemiology Reports 2 (2015): 221–228, 10.1007/s40471-015-0053-5. 26954351 PMC4778958

[36] C. T. Petersen , K. J. Jensen , M. Rosenzweig , et al., “Mapping Outcomes and Registries Used in Current Danish Pharmacoepidemiological Research,” Clinical Laboratory and Pharmacy 14 (2022): 521–542, 10.2147/CLEP.S341480. PMC9056023 35502197

[37] R. T. Birkett , E. Chamely , S. J. Concors , et al., “Overuse and Limited Benefit of Chemotherapy for Stage II Colon Cancer in Young Patients,” Clinical Colorectal Cancer 18 (2019): 292–300, 10.1016/j.clcc.2019.04.002. 31447135

[38] S. Nazzani , F. Preisser , E. Mazzone , et al., “Survival Effect of Perioperative Systemic Chemotherapy on Overall Mortality in Locally Advanced and/or Positive Regional Lymph Node Non‐Metastatic Urothelial Carcinoma of the Upper Urinary Tract,” World Journal of Urology 37 (2019): 1329–1337, 10.1007/s00345-018-2516-z. 30298285

[39] B. Babcock , M. Rodrigues , D. Kearns , et al., “Improved Survival With Immunotherapy but Lack of Synergistic Effect With Radiation for Stage IV Melanoma of the Head and Neck,” American Surgeon 85 (2019): 1118–1124, 10.1177/000313481908501009. 31657306

[40] E. S. Johnson , B. A. Bartman , B. A. Briesacher , et al., “The Incident User Design in Comparative Effectiveness Research,” Pharmacoepidemiology and Drug Safety 22 (2013): 1–6, 10.1002/pds.3334. 23023988

[41] E. Rotbain , H. Frederiksen , H. Hjalgrim , et al., “IGHV Mutational Status and Outcome for Patients With Chronic Lymphocytic Leukemia Upon Treatment: A Danish Nationwide Population‐Based Study,” Haematologica 105 (2020): 1621–1629. 31582540 10.3324/haematol.2019.220194 PMC7271602

[42] Q.‐H. Zhang , W.‐W. Zhang , J. Wang , et al., “Impact of the 21‐Gene Recurrence Score Assay on Chemotherapy Decision Making and Outcomes for Breast Cancer Patients With Four or More Positive Lymph Nodes,” Annals of Translational Medicine 7 (2019): 82. 31700882 10.21037/atm.2019.08.82 PMC6803245

[43] L. G. Hemkens , E. I. Benchimol , S. M. Langan , et al., “The Reporting of Studies Using Routinely Collected Health Data Was Often Insufficient,” Journal of Clinical Epidemiology 79 (2016): 104–111, 10.1016/j.jclinepi.2016.06.005. 27343981 PMC5152936

[44] Y. Jin , N. Sanger , I. Shams , et al., “Does the Medical Literature Remain Inadequately Described Despite Having Reporting Guidelines for 21 Years? – A Systematic Review of Reviews: An Update,” Journal of Multidisciplinary Healthcare 11 (2018): 495–510, 10.2147/JMDH.S155103. 30310289 PMC6166749

