# Supplementary material for: Methods Applied to Assess Real‐World Effectiveness of Drugs: A Scoping Review
Source: Pharmacol Res Perspect. 2026 May 5;14(3):e70244. doi: 10.1002/prp2.70244 (PMC13143864; doi:10.1002/prp2.70244)
Supplement: Supplementary file 1 — Table S1: Elaboration of the methods section. Table S2: PubMed search string and results as of 16MAR2022. Table S3: Embase search string and results as of 22MAR2022. Table S4: Data items collected and used in the data synthesis. Table S5: List of the 87 included studies. Table S6: Frequency of studies with specific ATC groups. Table S7: Key characteristics of the 87 included studies divided in studies investigating the effectiveness of chemotherapy and studies investigating the effectiveness of other drugs. [file PRP2-14-e70244-s001.docx]

# Methods applied to assess real-world effectiveness of drugs: A scoping review

**Supplementary information**

*von Osmanski et al*

[**Table S1: Elaboration of the methods section** 2](#_Toc200313295)

[**Table S2: PubMed search string and results as of 16MAR2022** 3](#_Toc200313296)

[**Table S3: Embase search string and results as of 22MAR2022** 4](#_Toc200313297)

[**Table S4: Data items collected and used in the data synthesis** 5](#_Toc200313298)

[**Table S5: List of the 87 included studies** 7](#_Toc200313299)

[**Table S6: Frequency of studies with specific ATC groups** 15](#_Toc200313300)

[**Table S7: Key characteristics of the 87 included studies divided by chemotherapy** 16](#_Toc200313301)

**Table S1: Elaboration of the methods section**

| **Section** | **Elaboration** |
| --- | --- |
| 2.1 Eligibility criteria: Exposure | Studies where non-drug exposures were investigated, but where a drug effect could be isolated from the remaining exposures, were considered eligible.  A drug administered in combination with a biological graft, e.g., a ligament graft soaked in antibiotics prior to implantation, were considered fulfilling the exposure definition, as we had not applied a criterion regarding route of drug administration. Following EMAs definition [1], drug-coated stents and other drug-containing devices were considered as medical devices and were not eligible for inclusion. |
| 2.3 Selection and data charting process: Title/abstract screening | The shift to single-reviewer screening was preceded by a validation process. In this process, two reviewers (CP and MA) each screened 150 records which were also screened by the third reviewer (BO). Of the 300 records screened in this process, 15 (5%) conflicts were detected. None of the conflict records, which were found eligible following discussion, had initially been excluded by reviewer BO. The protocol [2] states that all records should be double screened, but based on this evaluation, it was decided that the remaining records could be screened by one reviewer only (BO or MA). |
| 2.3 Selection and data charting process: Full text assessment | Five reviewers (BO, MA, JP, CP, and ES) took part in the first pilot phase, which was an iterative process where 25 studies were screened for final eligibility, and data were charted from included studies. The data charting was done using a formular in Microsoft Access designed a priori. Following individual screening and data charting, disagreements between reviewers were discussed and resolved, and adjustments to the formular were made. At the end of the first pilot phase all reviewers agreed on the final version of the formular, and the process proceeded to the second pilot phase which was conducted by three reviewers (BO, MA, and CP). In the second pilot phase, 28 studies were handled in three sessions. In the first two sessions all three reviewers screened the studies and charted data from included studies. Subsequently, the level of inter-reviewer agreement of the data charting was assessed by comparing the data items charted by the three reviewers, and disagreements were discussed. In the first two sessions, the level of agreement across the three reviewers was approx. 75%. As we considered it crucial to achieve a higher level of validity in our data, especially across the data items related to statistical methods, we decided to adjust our approach. Thus, in the final session of the second pilot phase, two reviewers (BO and CP) independently screened and charted data from the same studies, and, subsequently, the third reviewer (MA) validated the subset of data items related to statistical methods. In this session, the level of inter-reviewer agreement (prior to the statistical validation) was 84%, which was considered satisfactory. |

[1] European Medicines Agency (EMA) [homepage on the Internet]. Medical devices. November 26, 2018. Accessed January 20, 2025. https://www.ema.europa.eu/en/human-regulatory/overview/medical-devices

[2] von Osmanski BI, Ankarfeldt MZ, Petersen J, Petersen CT, Micheelsen A, Solem EJ. Available pharmacoepidemiological methods to assess real-world effectiveness of drugs: A scoping review protocol. Published online March 16, 2022. doi:10.17605/OSF.IO/XB2R5

**Table S2: PubMed search string and results as of 16MAR2022**

| **Search ID** | **Search terms** | **Number of records** |
| --- | --- | --- |
| #1 | "pharmacoepidemiology"[MeSH Terms] OR "pharmacoepidemiology"[Title/Abstract] OR "pharmacoepidemiologic"[Title/Abstract] OR "Observational"[Title/Abstract] OR "observational study"[Publication Type] OR "observational studies as topic"[MeSH Terms] OR "registry based"[Title/Abstract] OR "register based"[Title/Abstract] OR "registry study"[Title/Abstract] OR "register study"[Title/Abstract] OR "population based"[Title/Abstract] OR "real world study"[Title/Abstract] OR "epidemiologic studies"[MeSH Terms] OR "comparative effectiveness research"[MeSH Terms] OR "comparative study"[Publication Type] | 4,615,779 |
| #2 | "cross sectional studies"[MeSH Terms] OR "systematic review"[Publication Type] OR "Meta-analysis"[Publication Type] OR "Review"[Publication Type] | 3,489,262 |
| #3 | #1 NOT #2 | 4,019,978 |
| #4 | "registry"[Title/Abstract] OR "registries"[Title/Abstract] OR "registries"[MeSH Terms] OR "register"[Title/Abstract] OR "registers"[Title/Abstract] OR "database"[Title/Abstract] OR "databases"[Title/Abstract] OR "databases, factual"[MeSH Terms] OR "database management systems"[MeSH Terms] | 881,625 |
| #5 | "drug"[Title/Abstract] OR "drugs"[Title/Abstract] OR "drug therapy"[MeSH Terms] OR "drug therapy"[MeSH Subheading] OR "therapeutic use"[MeSH Subheading:noexp] OR "administration and dosage"[MeSH Subheading] OR "therapeutic uses"[MeSH Terms] | 6,091,783 |
| #6 | #3 AND #4 AND #5 | 58,234 |
| #7 | #6 limited to 01JUL2019-31DEC2019 | 3,303 |
| #8 | #7 limited to publications in English language | 3,277 |

**Table S3: Embase search string and results as of 22MAR2022**

| **Search ID** | **Search terms** | **Number of records** |
| --- | --- | --- |
| #1 | (registry or registries).tw,kw. | 258,554 |
| #2 | exp register/ | 178,507 |
| #3 | (register or registers).tw,kw. | 110,071 |
| #4 | (database or databases).tw,kw. | 819,336 |
| #5 | exp data base/ | 477,523 |
| #6 | exp database management system/ | 580 |
| #7 | 1 or 2 or 3 or 4 or 5 or 6 | 1,306,799 |
| #8 | exp drug therapy/ | 3,177,299 |
| #9 | (drug or drugs).tw,kw. | 2,455,314 |
| #10 | exp drug/ | 3,344,488 |
| #11 | exp drug exposure/ | 54,033 |
| #12 | exp drug administration/ | 1,223,989 |
| #13 | 8 or 9 or 10 or 11 or 12 | 8,290,421 |
| #14 | (pharmacoepidemiology or pharmacoepidemiologic).tw,kw. | 6,745 |
| #15 | observational.tw,kw. | 350,177 |
| #16 | exp observational study/ | 266,327 |
| #17 | exp observational method/ | 7,080 |
| #18 | (registry based or register based).tw,kw. | 9,241 |
| #19 | (registry study or register study).tw,kw. | 6,809 |
| #20 | population based.tw,kw. | 199,973 |
| #21 | real world study.tw,kw. | 3,976 |
| #22 | exp epidemiology/ | 3,978,241 |
| #23 | exp comparative study/ | 1,543,288 |
| #24 | 14 or 15 or 16 or 17 or 18 or 19 or 20 or 21 or 22 or 23 | 5,656,876 |
| #25 | exp cross-sectional study/ | 469,748 |
| #26 | review.pt. | 2,873,468 |
| #27 | conference abstract.pt. | 4,353,293 |
| #28 | 25 or 26 or 27 | 7,625,013 |
| #29 | 24 not 28 | 3,768,199 |
| #30 | 7 and 13 and 29 | 57,702 |
| #31 | limit 30 to english language | 55,678 |
| #32 | limit 31 to yr="2019" | 4,829 |
| #33 | limit 32 to dc=20190701-20191231 | 2,213 |

**Table S4: Data items collected and used in the data synthesis^a^**

|  | **Data item** | **Description** | **Coding** |
| --- | --- | --- | --- |
| **Publication characteris-tics** | Study ID | Each included study was given a unique study ID | Number |
|  | Author | First Author name + et al., eg, “Hansen, A.J. et al” | Text string |
|  | Title | Full publication title | Text string |
|  | Country | Country of data origin | Dynamic list of options |
| **Study population** | N | Number of patients in the study population | Number |
|  | Exposure name | Generic name of the drug exposure under investigation, eg, “apixaban” | Text string |
|  | Exposure ATC | ATC code of the drug exposure(s) under investigation | Text string |
|  | Comparator category | Comparator category | Options:   - Drug comparator - Non-use of exposure drug - Non-drug comparator (e.g., surgery or radiation) - Different dose/administration of the same drug |
|  | Comparator name | If the comparator was another drug, the name of the comparator drug was extracted | Text string |
|  | Comparator ATC | ATC code of the comparator drug(s)  *Note: As the ATC codes were not reported in the studies, they were looked up by the reviewer in the data charting process.* | Text string |
| **Study design** | New user design | Was a new user design been applied in the study? | Yes/No  *Note: Had to be stated explicitly in the study when extracted as “yes”* |
|  | Effectiveness outcome(s) (specific) | All effectiveness outcomes were extracted in text format | Text string |
|  | Outcome categories | All effectiveness outcomes extracted in the field above was categorized in prespecified categories.  If two or more outcomes were compatible with the same category, the category was only extracted once.  There were four fields for this item in the formular. | Options:   - Mortality/Survivial: All-cause - Mortality/Survival: Cause specific - Hospital admission: All-cause (includes admissions to ICU) - Hospital admission: Cause specific - Health care utilization (other than hospital admission. Includes length of hospitalization) - Drug prescription/discontinuation/switch* - Disease specific measures - Surgery and procedures - Costs: Overall - Costs: Disease/treatment specific - Diagnosis |
| **Statistical models** | Statistical model(s) (as reported) | Specification of the statistical model(s) applied in the study. Eg, Cox regression or negative binominal regression.  There were four fields for this item in the formular | Dynamic list of options |
|  | Confounder control | Eg, ‘Propensity score matching’ or ‘Inverse probability of treatment weights’  More than one option can be chosen.  Must be extracted in relation to the matching statistical model. Two confounder control fields are provided for each statistical model field in the formular. | Dynamic list of options |
| **Variables** | Variables | Which categories of variables were controlled for in the study? | Yes/no/unknown for each of the following categories:   - Social variables - Demographic variables - Comorbidity - Disease severity |

^a^ This table only includes data items used in our synthesis. More items were extracted.

# **Table S5: List of the 87 included studies**

| 1. Agra-Bermejo R, Cordero A, Rodríguez-Mañero M, et al. Clinical impact of mineralocorticoid receptor antagonists treatment after acute coronary syndrome in the real world: A propensity score matching analysis. *European heart journal Acute cardiovascular care*. 2019;8(7):652-659. doi:10.1177/2048872618795422  2. Aly A, Johnson C, Yang S, Botteman MF, Rao S, Hussain A. Overall survival, costs, and healthcare resource use by line of therapy in Medicare patients with newly diagnosed metastatic urothelial carcinoma. *Journal of medical economics*. 2019;22(7):662-670. doi:10.1080/13696998.2019.1591424  3. Alyafie F, Soliman AT, Sabt A, et al. Postnatal growth of Infants with neonatal diabetes: insulin pump (CSII) versus Multiple Daily Injection (MDI) therapy. *Acta bio-medica : Atenei Parmensis*. 2019;90(8):28-35. doi:10.23750/abm.v90i8-S.6719  4. Amin AA, Araj FG, Ariyamuthu VK, et al. Impact of induction immunosuppression on patient survival in heart transplant recipients treated with tacrolimus and mycophenolic acid in the current allocation era. *Clinical transplantation*. 2019;33(8):e13651. doi:10.1111/ctr.13651  5. Ann SH, Strauss MH, Park GM, et al. Comparison between angiotensin-converting enzyme inhibitor and angiotensin receptor blocker after percutaneous coronary intervention. *International journal of cardiology*. 2020;306:35-41. doi:10.1016/j.ijcard.2019.11.086  6. Anthony SG, Patterson DC, Cagle PJ Jr, et al. Utilization and Real-world Effectiveness of Tranexamic Use in Shoulder Arthroplasty: A Population-based Study. *The Journal of the American Academy of Orthopaedic Surgeons*. 2019;27(19):736-742. doi:10.5435/JAAOS-D-18-00206  7. Babcock B, Rodrigues M, Kearns D, et al. Improved Survival with Immunotherapy but Lack of Synergistic Effect with Radiation for Stage IV Melanoma of the Head and Neck. *The American surgeon*. 2019;85(10):1118-1124. doi:10.1177/000313481908501009  8. Bertero E, Miceli R, Lorenzoni A, et al. Causes and impact on survival of underuse of angiotensin-converting enzyme inhibitors and angiotensin II receptor blockers in heart failure. *Internal and emergency medicine*. 2019;14(7):1083-1090. doi:10.1007/s11739-019-02060-0  9. Birkett RT, Chamely E, Concors SJ, et al. Overuse and Limited Benefit of Chemotherapy for Stage II Colon Cancer in Young Patients. *Clinical colorectal cancer*. 2019;18(4):292-300. doi:10.1016/j.clcc.2019.04.002  10. Boel JB, Jansåker F, Hertz FB, et al. Treatment duration of pivmecillinam in men, non-pregnant and pregnant women for community-acquired urinary tract infections caused by Escherichia coli: a retrospective Danish cohort study. *The Journal of antimicrobial chemotherapy*. 2019;74(9):2767-2773.  11. Bonfanti L, Lippi G, Donelli V, Pigna F, Saccenti C, Cervellin G. Is anticoagulant therapy always indicated in “medium-risk” patients with first diagnosed atrial fibrillation? Insights from a real world, 10-year observational study. *International journal of cardiology*. 2019;288:76-81. doi:10.1016/j.ijcard.2019.04.057  12. Bunclark K, Newnham M, Chiu YD, et al. A multicenter study of anticoagulation in operable chronic thromboembolic pulmonary hypertension. *Journal of thrombosis and haemostasis : JTH*. 2020;18(1):114-122. doi:10.1111/jth.14649  13. Cañete F, Mañosa M, Casanova MJ, et al. Adalimumab or Infliximab for the Prevention of Early Postoperative Recurrence of Crohn Disease: Results From the ENEIDA Registry. *Inflammatory bowel diseases*. 2019;25(11):1862-1870. doi:10.1093/ibd/izz084  14. Carter KT, Lirette ST, Baran DA, et al. The Effect of Cardiac Preservation Solutions on Heart Transplant Survival. *The Journal of surgical research*. 2019;242:157-165. doi:10.1016/j.jss.2019.04.041  15. Casetta I, Pracucci G, Saletti A, et al. Combined intravenous and endovascular treatment versus primary mechanical thrombectomy. The Italian Registry of Endovascular Treatment in Acute Stroke. *International journal of stroke : official journal of the International Stroke Society*. 2019;14(9):898-907. doi:10.1177/1747493019851279  16. Catapano JS, Israr S, Whiting AC, et al. Management of Extracranial Blunt Cerebrovascular Injuries: Experience with an Aspirin-Based Approach. *World neurosurgery*. 2020;133:e385-e390. doi:10.1016/j.wneu.2019.09.013  17. Chan KKW, Guo H, Cheng S, et al. Real-world outcomes of FOLFIRINOX vs gemcitabine and nab-paclitaxel in advanced pancreatic cancer: A population-based propensity score-weighted analysis. *Cancer medicine*. 2020;9(1):160-169. doi:10.1002/cam4.2705  18. Chan YH, Lee HF, See LC, et al. Effectiveness and Safety of Four Direct Oral Anticoagulants in Asian Patients With Nonvalvular Atrial Fibrillation. *Chest*. 2019;156(3):529-543. doi:10.1016/j.chest.2019.04.108  19. Chen DY, Yu F, Tuan LW, Tang CH. Comparison of Healthcare Utilization and Costs Between RA Patients Receiving Biological and Conventional Synthetic DMARDs: A Nationwide Population-Based Cohort Study in Taiwan. *Frontiers in pharmacology*. 2019;10:1214. doi:10.3389/fphar.2019.01214  20. Chen G, Farris MS, Cowling T, et al. Treatment and Low-Density Lipoprotein Cholesterol Management in Patients Diagnosed With Clinical Atherosclerotic Cardiovascular Disease in Alberta. *The Canadian journal of cardiology*. 2019;35(7):884-891. doi:10.1016/j.cjca.2019.04.008  21. Chen YW, Voelker J, Tunceli O, Pericone CD, Bookhart B, Durkin M. Real-world comparison of hospitalization costs for heart failure in type 2 diabetes mellitus patients with established cardiovascular disease treated with canagliflozin versus other antihyperglycemic agents. *Journal of medical economics*. 2020;23(4):401-406. doi:10.1080/13696998.2019.1693384  22. Dalon F, Roche N, Belhassen M, et al. Dual versus triple therapy in patients hospitalized for COPD in France: a claims data study. *International journal of chronic obstructive pulmonary disease*. 2019;14:1839-1854. doi:10.2147/COPD.S214061  23. Dhamane AD, Baker CL, Rajpura J, et al. Continuation with apixaban treatment is associated with lower risk for hospitalization and medical costs among elderly patients. *Current medical research and opinion*. 2019;35(10):1769-1776. doi:10.1080/03007995.2019.1623187  24. Dharia I, Bielamowicz S. Unilateral versus bilateral botulinum toxin injections in adductor spasmodic dysphonia in a large cohort. *The Laryngoscope*. 2020;130(11):2659-2662. doi:10.1002/lary.28457  25. Elhai M, Boubaya M, Distler O, et al. Outcomes of patients with systemic sclerosis treated with rituximab in contemporary practice: a prospective cohort study. *Annals of the rheumatic diseases*. 2019;78(7):979-987. doi:10.1136/annrheumdis-2018-214816  26. Ermini G, Tosetti C, Zocchi D, Mandreoli M, Caletti MT, Marchesini G. Type 2 diabetes treatment and progression of chronic kidney disease in Italian family practice. *Journal of endocrinological investigation*. 2019;42(7):787-796. doi:10.1007/s40618-018-0983-0  27. Fields AC, Lu P, Goldberg J, Irani J, Bleday R, Melnitchouk N. The role of adjuvant chemotherapy in stage II and III mucinous colon cancer. *Journal of surgical oncology*. 2019;120(7):1190-1200. doi:10.1002/jso.25705  28. Firman B, Molnar A, Gray PH. Early high-dose caffeine citrate for extremely preterm infants: Neonatal and neurodevelopmental outcomes. *Journal of paediatrics and child health*. 2019;55(12):1451-1457. doi:10.1111/jpc.14446  29. Geng ZM, Cai ZQ, Zhang Z, et al. Estimating survival benefit of adjuvant therapy based on a Bayesian network prediction model in curatively resected advanced gallbladder adenocarcinoma. *World journal of gastroenterology*. 2019;25(37):5655-5666. doi:10.3748/wjg.v25.i37.5655  30. Glassberg MB, Lachiewicz PF. Changing Patterns of Anticoagulation After Total Hip Arthroplasty in the United States: Frequency of Deep Vein Thrombosis, Pulmonary Embolism, and Complications With Rivaroxaban and Warfarin. *The Journal of arthroplasty*. 2019;34(8):1793-1801. doi:10.1016/j.arth.2019.03.057  31. Harms MH, van Buuren HR, Corpechot C, et al. Ursodeoxycholic acid therapy and liver transplant-free survival in patients with primary biliary cholangitis. *Journal of hepatology*. 2019;71(2):357-365. doi:10.1016/j.jhep.2019.04.001  32. Hsu CY, Liu KD, Yang J, et al. Renin-Angiotensin System Blockade after Acute Kidney Injury (AKI) and Risk of Recurrent AKI. *Clinical journal of the American Society of Nephrology : CJASN*. 2020;15(1):26-34. doi:10.2215/CJN.05800519  33. Hung YC, Westfal ML, Chang DC, Kelleher CM. Heller myotomy is the optimal index procedure for esophageal achalasia in adolescents and young adults. *Surgical endoscopy*. 2019;33(10):3355-3360. doi:10.1007/s00464-018-06625-6  34. Jansson M, Själander S, Sjögren V, Renlund H, Norrving B, Själander A. Direct comparisons of effectiveness and safety of treatment with Apixaban, Dabigatran and Rivaroxaban in atrial fibrillation. *Thrombosis research*. 2020;185:135-141. doi:doi.org/10.1016/j.thromres.2019.11.010  35. Kalilani L, Faught E, Kim H, et al. Assessment and effect of a gap between new-onset epilepsy diagnosis and treatment in the US. *Neurology*. 2019;92(19):E2197-E2208. doi:10.1212/WNL.0000000000007448  36. Karavias D, Thomas P, Koh A, et al. Statin therapy does not influence the outcome of patients undergoing surgery for pancreatic cancer. *ANZ journal of surgery*. 2020;90(9):1671-1676. doi:10.1111/ans.15600  37. Kent DJ, McMahill-Walraven CN, Panozzo CA, et al. Descriptive Analysis of Long- and Intermediate-Acting Insulin and Key Safety Outcomes in Adults with Type 2 Diabetes Mellitus. *Journal of managed care & specialty pharmacy*. 2019;25(11):1162-1171. doi:10.18553/jmcp.2019.19042  38. Kim C, Duan L, Phan DQ, Lee MS. Frequency of Utilization of Beta Blockers in Patients With Heart Failure and Depression and Their Effect on Mortality. *The American journal of cardiology*. 2019;124(5):746-750. doi:10.1016/j.amjcard.2019.05.054  39. Kim D, Park J.-M, Kang K, et al. Dual Versus Mono Antiplatelet Therapy in Large Atherosclerotic Stroke: A Retrospective Analysis of the Nationwide Multicenter Stroke Registry. *Stroke*. 2019;50(5):1184-1192. doi:10.1161/STROKEAHA.119.024786  40. Kubo M, Kawai M, Kumamaru H, et al. A population-based recurrence risk management study of patients with pT1 node-negative HER2+ breast cancer: a National Clinical Database study. *Breast cancer research and treatment*. 2019;178(3):647-656. doi:10.1007/s10549-019-05413-7  41. LaVallee C, Cronin P, Bansal I, Kwong WJ, Boccia R. Importance of Initial Complete Parenteral Iron Repletion on Hemoglobin Level Normalization and Health Care Resource Utilization: A Retrospective Analysis. *Pharmacotherapy*. 2019;39(10):983-993. doi:10.1002/phar.2319  42. Legué LM, van Erning FN, Bernards N, Lemmens VEPP, de Hingh IHJT, Creemers GJ. Addition of Bevacizumab to First-Line Palliative Chemotherapy in Patients with Metastatic Small Bowel Adenocarcinoma: A Population-Based Study. *Targeted oncology*. 2019;14(6):699-705. doi:10.1007/s11523-019-00681-1  43. Li X, Zhang C, Sun Z, et al. Propensity-matched analysis of adjuvant chemotherapy for completely resected Stage IB non-small-cell lung cancer patients. *Lung cancer (Amsterdam, Netherlands)*. 2019;133:75-82. doi:10.1016/j.lungcan.2019.04.024  44. Liu WN, Chang CF, Chung CH, et al. Clinical outcomes of bortezomib-based therapy in Taiwanese patients with multiple myeloma: A nationwide population-based study and a single-institute analysis. *PloS one*. 2019;14(9):e0222522. doi:10.1371/journal.pone.0222522  45. Liu WX, Shi M, Su H, et al. Effect of age as a continuous variable on survival outcomes and treatment selection in patients with extranodal nasal-type NK/T-cell lymphoma from the China Lymphoma Collaborative Group (CLCG). *Aging*. 2019;11(19):8463-8473. doi:10.18632/aging.102331  46. Lu-Yao G, Nikita N, Keith SW, et al. Mortality and Hospitalization Risk Following Oral Androgen Signaling Inhibitors Among Men with Advanced Prostate Cancer by Pre-existing Cardiovascular Comorbidities. *European urology*. 2020;77(2):158-166. doi:10.1016/j.eururo.2019.07.031  47. Margalit O, Mamtani R, Kopetz S, et al. Refining the Use of Adjuvant Oxaliplatin in Clinical Stage II or III Rectal Adenocarcinoma. *The oncologist*. 2019;24(8):e671-e676. doi:10.1634/theoncologist.2018-0333  48. Matusevicius M, Paciaroni M, Caso V, et al. Outcome after intravenous thrombolysis in patients with acute lacunar stroke: An observational study based on SITS international registry and a meta-analysis. *International journal of stroke : official journal of the International Stroke Society*. 2019;14(9):878-886. doi:10.1177/1747493019840947  49. McClelland S, Hatfield J, Degnin C, Chen Y, Mitin T. Extent of resection and role of adjuvant treatment in resected localized breast angiosarcoma. *Breast Cancer Research and Treatment*. 2019;175(2):409-418. doi:10.1007/s10549-019-05172-5  50. McDow AD, Shumway CM, Pitt SC, Schneider DF, Sippel RS, Long KL. Utility of Early Postoperative Unstimulated Thyroglobulin in Influencing Decision Making in Patients with Papillary Thyroid Carcinoma. *Annals of surgical oncology*. 2019;26(12):4002-4007. doi:10.1245/s10434-019-07581-8  51. Meers S, Bailly B, Vande Broek I, et al. Real-world data confirming the efficacy and safety of decitabine in acute myeloid leukaemia - results from a retrospective Belgian registry study. *Acta clinica Belgica*. 2021;76(2):98-105. doi:10.1080/17843286.2019.1665233  52. Moretz C, Bengtson LG, Sharpsten L, et al. Evaluation of rescue medication use and medication adherence receiving umeclidinium/vilanterol versus tiotropium bromide/olodaterol. *International journal of chronic obstructive pulmonary disease*. 2019;14:2047-2060. doi:10.2147/COPD.S213520  53. Morrison VA, Hamilton L, Ogbonnaya A, Raju A, Hennenfent K, Galaznik A. Treatment approaches for older and oldest patients with diffuse large B-cell lymphoma - Use of non-R-CHOP alternative therapies and impact of comorbidities on treatment choices and outcome: A Humedica database retrospective cohort analysis, 2007-2015. *Journal of geriatric oncology*. 2020;11(1):41-54. doi:10.1016/j.jgo.2019.07.025  54. Morvan AC, Hengy B, Garrouste-Orgeas M, et al. Impact of species and antibiotic therapy of enterococcal peritonitis on 30-day mortality in critical care-an analysis of the OUTCOMEREA database. *Critical care (London, England)*. 2019;23(1):307. doi:10.1186/s13054-019-2581-8  55. Mothojakan NB, Gore J, Nisar MK. Does biologic survival depend on co-prescribed methotrexate dose in established rheumatoid arthritis? A real-world study. *European journal of rheumatology*. 2020;7(1):21-25. doi:10.5152/eurjrheum.2019.19048  56. Munoz-Zuluaga CA, King MC, Ledakis P, et al. Systemic chemotherapy before cytoreductive surgery and hyperthermic intraperitoneal chemotherapy (CRS/HIPEC) in patients with high-grade mucinous carcinoma peritonei of appendiceal origin. *European journal of surgical oncology : the journal of the European Society of Surgical Oncology and the British Association of Surgical Oncology*. 2019;45(9):1598-1606. doi:10.1016/j.ejso.2019.05.008  57. Nakashima M, Ide K, Kawakami K. Comparison of Standard Initial Dose and Reduced Initial Dose Regorafenib for Colorectal Cancer Patients: A Retrospective Cohort Study. *Targeted Oncology*. 2019;14(3):295-306. doi:10.1007/s11523-019-00642-8  58. Narvaez JRF, Noyes K, Nie J, Kayler LK. Outcomes of DCD kidneys recovered for transplantation with versus without pre-mortem heparin administration. *Clinical transplantation*. 2019;33(7):e13624. doi:10.1111/ctr.13624  59. Nasioudis D, Latif NA, Simpkins F, et al. Adjuvant chemotherapy for early stage endometrioid ovarian carcinoma: An analysis of the National Cancer Data Base. *Gynecologic oncology*. 2020;156(2):315-319. doi:10.1016/j.ygyno.2019.11.125  60. Nazzani S, Preisser F, Mazzone E, et al. Survival effect of perioperative systemic chemotherapy on overall mortality in locally advanced and/or positive regional lymph node non-metastatic urothelial carcinoma of the upper urinary tract. *World journal of urology*. 2019;37(7):1329-1337. doi:10.1007/s00345-018-2516-z  61. O’Halloran N, Lowery A, Curran C, et al. A Review of the Impact of Neoadjuvant Chemotherapy on Breast Surgery Practice and Outcomes. *Clinical breast cancer*. 2019;19(5):377-382. doi:10.1016/j.clbc.2019.04.011  62. Parmar GM, Novak Z, Spangler E, et al. Statin use improves limb salvage after intervention for peripheral arterial disease. *Journal of vascular surgery*. 2019;70(2):539-546. doi:10.1016/j.jvs.2018.07.089  63. Pierone G, Henegar C, Fusco J, et al. Two-drug antiretroviral regimens: an assessment of virologic response and durability among treatment-experienced persons living with HIV in the OPERA(®) Observational Database. *Journal of the International AIDS Society*. 2019;22(12):e25418. doi:10.1002/jia2.25418  64. Rosiello G, Knipper S, Palumbo C, et al. Increasing Rates of Perioperative Chemotherapy are Associated With Improved Survival in Men With Urothelial Bladder Cancer With Prostatic Stromal Invasion. *Clinical genitourinary cancer*. 2020;18(1):35-44.e1. doi:10.1016/j.clgc.2019.10.012  65. Rotbain EC, Frederiksen H, Hjalgrim H, et al. IGHV mutational status and outcome for patients with chronic lymphocytic leukemia upon treatment: a Danish nationwide population-based study. *Haematologica*. 2020;105(6):1621-1629. doi:10.3324/haematol.2019.220194  66. Santamarina E, Parejo Carbonell B, Sala J, et al. Use of intravenous brivaracetam in status epilepticus: A multicenter registry. *Epilepsia*. 2019;60(8):1593-1601. doi:10.1111/epi.16094  67. Saraswathula A, Chen MM, Colevas AD, Divi V. Assessing Care Value for Older Patients Receiving Radiotherapy With or Without Cisplatin or Cetuximab for Locoregionally Advanced Head and Neck Cancer. *JAMA otolaryngology-- head & neck surgery*. 2019;145(12):1160-1167. doi:10.1001/jamaoto.2019.2381  68. Shao SC, Chang KC, Hung MJ, et al. Comparative risk evaluation for cardiovascular events associated with dapagliflozin vs. empagliflozin in real-world type 2 diabetes patients: a multi-institutional cohort study. *Cardiovascular diabetology*. 2019;18(1):120. doi:10.1186/s12933-019-0919-9  69. Shigemi D, Matsui H, Fushimi K, Yasunaga H. Therapeutic Impact of Initial Treatment for Chlamydia trachomatis Among Patients With Pelvic Inflammatory Disease: A Retrospective Cohort Study Using a National Inpatient Database in Japan. *Clinical infectious diseases : an official publication of the Infectious Diseases Society of America*. 2019;69(2):316-322. doi:10.1093/cid/ciy862  70. Singh SB, McLearn-Montz AJ, Milavetz F, et al. Pathogen acquisition in patients with cystic fibrosis receiving ivacaftor or lumacaftor/ivacaftor. *Pediatric pulmonology*. 2019;54(8):1200-1208. doi:10.1002/ppul.24341  71. Strain W.D, McEwan P, Howitt H, Meadowcroft S. Retrospective Database Analysis Evaluating the Clinical Outcomes of Changing Treatment of People with Type 2 Diabetes Mellitus (T2DM) from Other DPP-4 Inhibitor Therapy to Alogliptin in a Primary Care Setting. *Diabetes Therapy*. 2019;10(4):1499-1507. doi:10.1007/s13300-019-0662-y  72. Strati P, Abdelrahim M, Selamet U, et al. Ruxolitinib therapy is associated with improved renal function in patients with primary myelofibrosis. *Annals of hematology*. 2019;98(7):1611-1616. doi:10.1007/s00277-019-03708-9  73. Syeda S, Chen L, Hou J.Y, et al. Chemotherapy, Radiation, or Combination Therapy for Stage III Uterine Cancer. *Obstetrics and Gynecology*. 2019;134(1):17-29. doi:10.1097/AOG.0000000000003287  74. Thomson J, Hall M, Ambroggio L, et al. Antibiotics for Aspiration Pneumonia in Neurologically Impaired Children. *Journal of hospital medicine*. 2020;15(7):395-402. doi:10.12788/jhm.3338  75. Torres AD, Sparvoli JMH, Sparvoli AC, Gonçalves CV. Sustained virologic response rate in chronic hepatitis C patients through direct-acting antivirals therapy. *Arquivos de gastroenterologia*. 2019;56(4):394-398. doi:10.1590/S0004-2803.201900000-79  76. Tran TB, Maker VK, Maker AV. Impact of Immunotherapy after Resection of Pancreatic Cancer. *Journal of the American College of Surgeons*. 2019;229(1):19-27.e1. doi:10.1016/j.jamcollsurg.2019.01.016  77. Truby LK, Farr MA, Garan AR, et al. Impact of Bridge to Transplantation With Continuous-Flow Left Ventricular Assist Devices on Posttransplantation Mortality. *Circulation*. 2019;140(6):459-469. doi:10.1161/CIRCULATIONAHA.118.036932  78. Tsutsumi I, Kunisawa S, Yoshida C, et al. Impact of oral voriconazole during chemotherapy for acute myeloid leukemia and myelodysplastic syndrome: a Japanese nationwide retrospective cohort study. *International journal of clinical oncology*. 2019;24(11):1449-1458. doi:10.1007/s10147-019-01506-x  79. van Putten M, Lemmens VEPP, van Laarhoven HWM, Pruijt HFM, Nieuwenhuijzen GAP, Verhoeven RHA. Poor compliance with perioperative chemotherapy for resectable gastric cancer and its impact on survival. *European journal of surgical oncology : the journal of the European Society of Surgical Oncology and the British Association of Surgical Oncology*. 2019;45(10):1926-1933. doi:10.1016/j.ejso.2019.03.040  80. Wakeam E, Adibfar A, Stokes S, et al. Defining the role of adjuvant therapy for early-stage large cell neuroendocrine carcinoma. *The Journal of thoracic and cardiovascular surgery*. 2020;159(5):2043-2054.e9. doi:10.1016/j.jtcvs.2019.09.077  81. Wu J, Chen Y, Hageman L, et al. Late mortality after bone marrow transplant for chronic myelogenous leukemia in the context of prior tyrosine kinase inhibitor exposure: A Blood or Marrow Transplant Survivor Study (BMTSS) report. *Cancer*. 2019;125(22):4033-4042. doi:10.1002/cncr. 324 43  82. Wyles CC, Vargas-Hernandez JS, Carlson SW, Carlson BC, Sierra RJ. Single-Dose Perioperative Antibiotics Do Not Increase the Risk of Surgical Site Infection in Unicompartmental Knee Arthroplasty. *The Journal of arthroplasty*. 2019;34(7):S327-S330. doi:10.1016/j.arth.2019.02.041  83. Xiang M, Kidd E.A. Benefit of cisplatin with definitive radiotherapy in older women with cervical cancer. *JNCCN Journal of the National Comprehensive Cancer Network*. 2019;17(8):969-975. doi:10.6004/jnccn.2019.7289  84. Zhang Q.-H, Zhang W.-W, Wang J, et al. Impact of the 21-gene recurrence score assay on chemotherapy decision making and outcomes for breast cancer patients with four or more positive lymph nodes. *Annals of Translational Medicine*. 2019;7(18):82. doi:10.21037/atm.2019.08.82  85. Zhu F, Piotin M, Steglich-Arnholm H, et al. Periprocedural Heparin During Endovascular Treatment of Tandem Lesions in Patients with Acute Ischemic Stroke: A Propensity Score Analysis from TITAN Registry. *Cardiovascular and interventional radiology*. 2019;42(8):1160-1167. doi:10.1007/s00270-019-02251-4  86. Zongo A, Simpson S, Johnson JA, Eurich DT. Optimal threshold of adherence to lipid lowering drugs in predicting acute coronary syndrome, stroke, or mortality: A cohort study. *PloS one*. 2019;14(9):e0223062. doi:10.1371/journal.pone.0223062  87. Zotzmann V, Rilinger J, Lang CN, et al. Epinephrine, inodilator, or no inotrope in venoarterial extracorporeal membrane oxygenation implantation: a single-center experience. *Critical care (London, England)*. 2019;23(1):320. doi:10.1186/s13054-019-2605-4 |
| --- |

**Table S6: Frequency of studies with specific ATC groups**

| ATC | Frequency  (n=94)^a^ |
| --- | --- |
| A: Alimentary tract and metabolism  A05: Bile and liver therapy  A07: Antidiarrheals, intestinal, anti-inflammatory/antiinfective agents  A10: Drugs used in diabetes | 1  1  6 |
| B: Blood and blood forming organs  B01: Antithrombotic agents  B02: Antihemorrhagics  B03: Antianemic preparations | 13  1  2 |
| C: Cardiovascular system  C01: Cardiac therapy  C03: Diuretics  C07: Beta blocking agents  C09: Agents acting on the renin-angiotensin system  C10: Lipid modifying agents | 1  1  1  3  3 |
| H: Systemic hormonal preparations, excl. sex hormones and insulins  H02: Corticosteroids for systemic use | 1 |
| J: Antiinfectives for systemic use  J01: Antibacterials for systemic use  J02: Antimycotics for systemic use  J05: Antivirals for systemic use | 5  1  2 |
| L: Antineoplastic and immunomodulating agents  L01: Antineoplastic agents  L02: Endocrine therapy  L04: Immunosuppressants | 32  1  6 |
| M: Musculo-skeletal system  M01: Antiinflammatory and antirheumatic products  M03: Muscle relaxants | 1  2 |
| N: Nervous system  N03: Antiepileptics  N06: Psychoanaleptics | 2  1 |
| P: Antiparasitic products, insecticides and repellents  P01: Antiprotozoals | 1 |
| R: Respiratory system  R03: Drugs for obstructive airway diseases  R07: Other respiratory system products | 2  1 |
| V: Various  V10: Therapeutic radiopharmaceuticals | 1 |
| Unknown ATC | 2 |

^a^Because more than one drug could be extracted from each study the numbers sum up to more than the 87 included studies. Each ATC group could only be counted once per study.

**Table S7: Key characteristics of the 87 included studies divided in studies investigating the effectiveness of chemotherapy and studies investigating the effectiveness of other drugs**

| Study characteristics | Studies with no chemo exposure (n=55), n (%) | Studies with chemo exposure^a^  (n=32), n (%) |
| --- | --- | --- |
| Area of data origin  US  Europe  Asia  Other  Multinational | 25 (45.5)  15 (27.3)  8 (14.6  4 (7.3)  3 (5.5) | 19 (59.4)  6 (18.8)  5 (15.6)  1 (3.1)  1 (3.1) |
| Number of patients  1-100  101-1,000  1001-10,000  10.001-100,000  > 100,000 | 5 (9.1)  15 (27.3)  16 (29.1)  16 (29.1)  3 (5.5) | 1 (3.1)  11 (34.4)  17 (53.1)  3 (9.4)  0 (0.0) |
| Comparator category (N=93)^b^  Non-use of exposure drug  Drug comparator  Different dose/admin. of the same drug  Non-drug comparator | 58  21 (36.2)  26 (44.8)  8 (13.8)  3 (5.2) | 35  21 (60.0)  9 (25.7)  3 (8.6)  2 (5.7) |
| New user design  No  Yes | 40 (72.7)  15 (27.3) | 25 (78.1)  7 (21.9) |
| Outcome category (N=178)^b^  Mortality/survival: All-cause  Disease specific measures  Diagnosis  Surgery and procedures  Health care utilization  Mortality/survival: Cause specific  Hospital admission: All-cause  Drug prescription/discontinuation/switch  Costs: Overall  Hospital admission: Cause specific  Costs: Disease/treatment specific | 122  24 (19.7)  25 (20.5)  17 (13.9)  16 (13.1)  9 (7.4)  4 (3.3)  6 (4.9)  6 (4.9)  4 (3.3)  6 (4.9)  5 (4.1) | 54  28 (51.9)  4 (7.4)  5 (9.3)  1 (1.9)  3 (5.6)  6 (11.1)  3 (5.6)  2 (3.7)  2 (3.7)  0 (0.0)  0 (0.0) |
| Statistical model category (N=97)^b^  Survival models  Regression models  Other models | 62  26 (41.9)  21 (33.9)  15 (24.2) | 34  29 (85.3)  4 (11.8)  1 (2.9) |
| Confounder control methods (N=138)^b^  Adjustment  Stratification  Propensity score matching  Inverse probability of treatment weighting (IPTW)  Matching  Propensity score adjustment  Propensity score stratification  Stabilized inverse probability treatment weighting  Full optimal matching  High-dimensional propensity score model with IPTW  Instrumental variable  Standardized mortality ratio weighting | 79  30 (38.0)  17 (21.5)  12 (15.2)  8 (10.1)  2 (2.5)  3 (3.8)  1 (1.3)  2 (2.5)  1 (1.3)  1 (1.3)  1 (1.3)  1 (1.3) | 58  22 (37.9)  19 (32.8)  10 (17.2)  5 (8.6)  1 (1.7)  0 (0.0)  1 (1.7)  0 (0.0)  0 (0.0)  0 (0.0)  0 (0.0)  0 (0.0) |
| Number of studies controlling for  Demographic variables  Comorbidity  Disease severity  Social variables | 40 (72.7)  32 (58.2)  31 (56.4)  8 (14.6) | 27 (84.4)  16 (50.0)  25 (78.1)  11 (34.4) |

^a^ Studies with at least one exposure from the L01-group (antineoplastic agents) in the Anatomical Therapeutic Chemical (ATC) classification system; ^b^ Because more than one category could be extracted from each study the numbers sum up to more than the 87 included studies. Each category could only be counted once per study.

Abbreviations: IPTW: inverse probability of treatment weighting.
